# The benefits of the digital chest drainage after pleuro - decortication in empyema. Prospective, comparative randomized trial

**DOI:** 10.1186/1749-8090-10-S1-A103

**Published:** 2015-12-16

**Authors:** José M Mier, G Cortés, A Berrios

**Affiliations:** 1 Thoracic Surgery Department National Institute of Respiratory Diseases INER México City, Mexico

## Background/Introduction

Persistent air leaks represent the most common pulmonary complication after empyema-pleuro-decortication. Since there are insufficient data in the literature regarding variability in the withdrawal of postoperative pleural drainages in pleuro-decortication.

## Aims/Objectives

We have designed a prospective, comparative and randomized study to evaluate if the use of digital chest drainage (Thopaz- Medela) to measure postoperative air leak compared to a classic device varies on deciding when to withdraw chest tubes and it diminished the complications and reoperation after pleuro-decortication.

## Method

A prospective, comparative and randomized trial was conducted in 37 patients who underwent pleuro-decortication in a empyema. Since May-Dec 2014. Male 27, female 10; mean-age 48years. We compared the use of digitals devices with the current analogue version. The digital and analogue group had 18 and 19 patients, respectively. The surgery was indicated when the empyema was 5-7grade (Light Classification). The measure of the air leak in the digital group was in ml/min, in the analogic group we perform the conversion between the bubbles scale (0-5) to ml/min. We compare the number of complication and reoperation cases in both groups and the day to withdraw the chest tube.

## Results

Clinical population data and Light Classification were not statistically different between the groups. Thoracotomy approach 94.6%, VATS 5.4%. The immediate postoperative air leak was in the 96% of the patients. The withdrawal of the chest tube in the Digital group 4.5 days; analogic 5.5 days (p = 0.49). The postoperative complication between digital and analogic groups were 22.2% vs 36.8% (p = 0.37). The reintervention was necessary in 16.67% vs 26.31% (p = 0.09)

## Discussion/Conclusion

The use of digital chest drainage in pleuro-decortication reduce the reoperation cases. We observe a tendency to reduce the air leak and the chest tube necessity in the digital group, but probably we need a large series for confirm this point.

